# Point-of-Care Ultrasound–Guided Management of New-Onset Atrial Flutter With Undiagnosed HFrEF and Transient Pericardial Effusion

**DOI:** 10.51894/001c.165691

**Published:** 2026-07-31

**Authors:** John Martinez, Anthony Costa, Sydney Moriarty, Sabrina Bahu, Garrett Beck

**Affiliations:** 1 Henry Ford - Genesys

## 98

### Introduction

Atrial flutter with rapid ventricular response (RVR) is commonly managed with beta blockers or non-dihydropyridine calcium channel blockers for rate control. However, treatment choice depends on underlying left ventricular systolic function, as calcium channel blockers are contraindicated in heart failure with reduced ejection fraction (HFrEF) due to negative inotropic effects. In acute settings where formal echocardiography may not be immediately available, point-of-care ultrasound (POCUS) can rapidly assess cardiac function and guide management. We present a case in which early POCUS identified previously undiagnosed HFrEF in a patient with atrial flutter, directly influencing treatment decisions.

### Case Presentation

A 65-year-old man with untreated hypertension, obstructive sleep apnea, class III obesity, and a 40-pack-year smoking history presented with progressive dyspnea, orthopnea, and severe edema. Vital signs showed blood pressure 154/114 mmHg and heart rate 156 bpm. Examination revealed anasarca, bilateral crackles, and tachycardia. Electrocardiogram demonstrated typical 2:1 atrial flutter with RVR. Laboratory testing showed BNP 3322 pg/mL and elevated high-sensitivity troponin with downtrend. Chest radiography demonstrated pulmonary congestion with bilateral pleural effusions. Bedside POCUS revealed bilateral B-lines, a dilated left ventricle with markedly reduced systolic function, and a small pericardial effusion. Concern for undiagnosed HFrEF prompted avoidance of non-dihydropyridine calcium channel blockers. Rate control was achieved with intravenous metoprolol followed by oral metoprolol and digoxin, while aggressive diuresis and anticoagulation were initiated. Formal echocardiography the following day confirmed an LVEF of 30–35% with mild right ventricular dysfunction and no pericardial effusion, suggesting the initial effusion was transient and related to volume overload.

### Discussion

This case highlights the role of POCUS in the early evaluation of tachyarrhythmias and undifferentiated dyspnea. Rapid bedside identification of systolic dysfunction allowed avoidance of potentially harmful therapy and supported targeted decongestive management. Integrating POCUS into early clinical assessment can improve risk stratification and guide safer, evidence-based treatment before formal imaging is available.

